# Admission Neutrophil-to-Lymphocyte Ratio and History of Suicide Attempt in Schizophrenia/Schizoaffective Disorder: A Retrospective Cohort (Israel, 2018–2024)

**DOI:** 10.1192/j.eurpsy.2026.12075

**Published:** 2026-07-31

**Authors:** H. Ghanaiem

**Affiliations:** Psychiatry, Yehuda Abarbanel Mental Health Center, Bat Yam, Israel

## Abstract

**Introduction:**

Neutrophil-to-lymphocyte ratio (NLR) is a readily available marker of systemic inflammation and has been linked to psychopathology and suicidality. In schizophrenia and schizoaffective disorder, objective indicators that aid suicide-risk stratification at admission are needed. We assessed whether admission NLR differs between patients with and without a documented history of suicide attempt in an Israeli psychiatric center.

**Objectives:**

To test whether NLR at admission/initial assessment differentiates patients with vs. without a documented history of suicide attempt.

**Methods:**

Design: Retrospective cohort at Yehuda Abarbanel Mental Health Center (2018–2024).

Participants: Adults with ICD-10 F20/F25 (schizophrenia/schizoaffective disorder).

Exposure: NLR from first complete blood count during index hospitalization.

Groups: Attempt+ (history of suicide attempt) vs. Attempt− (no history).

Statistics: Mann–Whitney U test, Hodges–Lehmann median difference (Δ), rank-biserial r; validation via Welch t-test on log(NLR).

**Results:**

Sample: N=103 (Attempt+ n=61; Attempt− n=42).

NLR (median [IQR]): Attempt+ 0.87 [0.47–1.59] vs. Attempt− 0.54 [0.35–0.67].

Mann–Whitney U=1731.5, p=0.0025; Hodges–Lehmann Δ=0.28; rank-biserial r≈0.35.

Log-transformed NLR: Welch t=3.42, p=0.0009 (~62.5% higher in Attempt+).

Age (years, median [IQR]): Attempt+ 45.0 [37.0–62.0] vs. Attempt− 38.0 [32.0–42.75]; p=0.0008.

**Conclusions:**

In this single-center retrospective cohort, admission NLR was significantly higher among inpatients with a prior suicide attempt than among those without, with a small–moderate effect size. These findings support NLR as an inexpensive, readily available adjunct, not a stand-alone test, for suicide-risk stratification at admission. Replication and prospective validation (including thresholding and clinical utility) are warranted.

**Disclosure of Interest:**

None Declared

